# Supplementary material for: Complex‐centric proteome profiling by SEC‐SWATH‐MS
Source: Mol Syst Biol. 2019 Jan 14;15(1):e8438. doi: 10.15252/msb.20188438 (PMC6346213; doi:10.15252/msb.20188438)
Supplement: Supplementary file 8 — Dataset EV7 [file MSB-15-e8438-s008.zip › feature_plots_string/Q9UHI6.pdf]

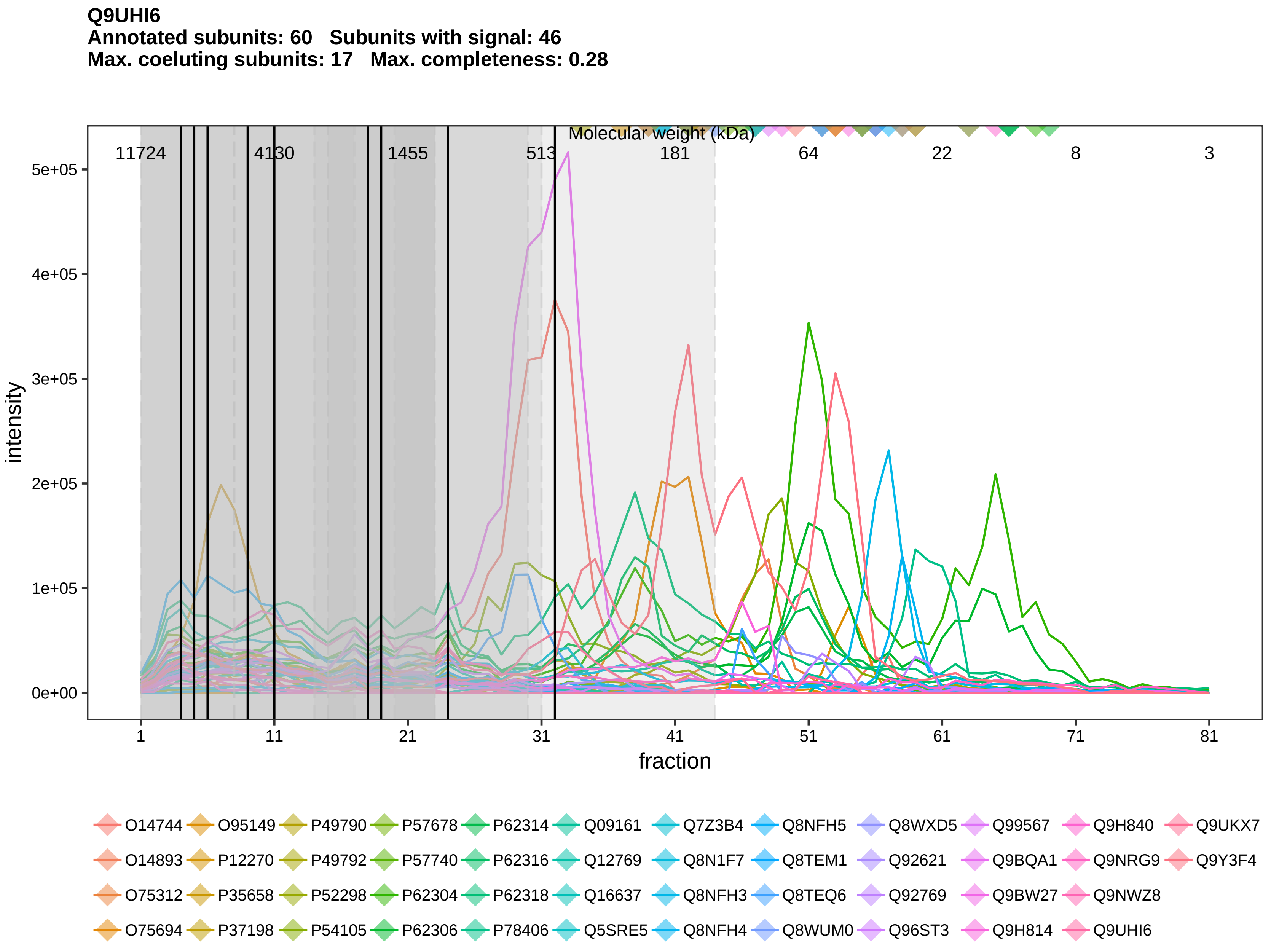
Q9UHI6
Annotated subunits: 60 Subunits with signal: 46
Max. coeluting subunits: 17 Max. completeness: 0.28
Molecular weight (kDa)
11724
4130
1455
513
181
64
22
8
3
intensity
5e+05
4e+05
3e+05
2e+05
1e+05
0e+00
fraction
1
11
21
31
41
51
61
71
81
O14744
O95149
P49790
P57678
P62314
Q09161
Q7Z3B4
Q8NFH5
Q8WXD5
Q99567
Q9H840
Q9UKX7
O14893
P12270
P49792
P57740
P62316
Q12769
Q8N1F7
Q8TEM1
Q92621
Q9BQA1
Q9NRG9
Q9Y3F4
O75312
P35658
P52298
P62304
P62318
Q16637
Q8NFH3
Q8TEQ6
Q92769
Q9BW27
Q9NWZ8
O75694
P37198
P54105
P62306
P78406
Q5SRE5
Q8NFH4
Q8WUM0
Q96ST3
Q9H814
Q9UHI6
